# Composite indices of femoral neck strength predicts the collapse of steroid-associated osteonecrosis of the femoral head: a retrospective study

**DOI:** 10.1186/s12891-022-05622-0

**Published:** 2022-07-28

**Authors:** Tianye Lin, Kaishen Cai, Peng Yang, Shana WuRi, Weijian Chen, Pan Deng, Ziqi Li, Zhenqiu Chen, Wei He, Qingwen Zhang, Qiushi Wei

**Affiliations:** 1 Joint Center, the Third Affiliated Hospital, Guangzhou University of Chinese Medicine, Guangzhou, Guangdong 510405 China; 2 Guangdong Research Institute for Orthopedics & Traumatology of Chinese Medicine, Guangzhou, Guangdong 510405 China; 3 The First Clinical Medical College of Guangzhou University of Chinese Medicine, Guangzhou, Guangdong 510405 China; 4 Guangzhou Orthopedic Hospital, Guangzhou University of Chinese Medicine, Guangzhou, Guangdong 510405 China; 5 Department of Joint Orthopaedic, Baoji Hospital of Traditional Chinese Medicine, Baoji, Shaanxi 721000 China; 6 The First Affiliated Hospital of Guangzhou University of Chinese Medicine, Guangzhou, Guangdong 510405 China

**Keywords:** Steroid-associated Osteonecrosis of the Femoral Head, Prediction, Collapse, Impact strength index, Bone turnover marker

## Abstract

**Purposes:**

The purpose of this study was to investigate the predictive effect exerted by composite indices of femoral neck strength (compressive strength index (CSI), bending strength index (BSI) and impact strength index (ISI) on the femoral head collapse in steroid-associated ONFH patients.

**Methods:**

Nonoperative steroid-associated osteonecrosis of the femoral head (ONFH) patients from 2017 to 2019 were selected. The patients fell into the collapsed group and the non-collapsed group according to whether the femoral head collapsed. CSI, BSI and ISI were calculated. Moreover, bone turnover markers were measured. The statistical analysis was conducted on the predictive effects of composite indices of femoral neck strength and bone turnover index on ONFH collapse.

**Results:**

A total of 62 patients were included. The mean CSI, BSI and ISI were significantly lower in the collapsed group than those in the non-collapsed group (*P* < 0.05). CSI, ISI,t-P1NP and β-CTx were suggested as the protective risk factors for the femoral head collapse in ONFH patients. The ISI area under the curve values was 0. 878.The mean survival time of the hips of patients with ISI greater than 0.435 was greater (*P* < 0.05) than that of patients with ISI less than 0.435.

**Conclusion:**

The composite indices of femoral neck strength can predict steroid-associated ONFH femoral head collapse more effectively than the bone turnover markers. The ISI value of 0.435 is a potential cut-off value, lower than this value can predict the early collapse of steroid-associated ONFH.

## Introduction

Osteonecrosis of the femoral head (ONFH), i.e., avascular necrosis of the femoral head, refers to a multifactorial disabling disease [1]. Corticosteroid are used clinically, and they are mostly used to treat allergic diseases and autoimmune diseases [2, 3]. Steroid-associated ONFH is one of the most common complications of high-dose corticosteroid use. With the widespread use of corticosteroid, the incidence of Steroid-associated ONFH is getting higher and higher. There are 8.12 million non-traumatic osteonecrosis patients in China alone [4]. It is estimated that there are 20,000 novel cases of ONFH in the United States each year, and the cumulative number of ONFH patients is 300,000 to 600,000 [5]. Femoral head necrosis progresses rapidly, and the disability rate is high. The research found 94% of early asymptomatic ONFH without treatment will advance as middle or late ONFH in 5 years [6]. When the disease progresses to the terminal stage, the femoral head is flat, and the joint space is severely narrowed. The appearance of the femoral head collapse indicates a poor prognosis for ONFH. The femoral head collapse may be related to biological and biomechanical factors [7]. The collapse of the femoral head is related to numerous factors (e.g., the size of the necrotic area, the shape of the necrosis). However, no effective indicator has been developed to predict ONFH collapse.

The use of high-dose corticosteroid can also cause bone loss. Glucocorticoids prolong the survival time of osteoclasts, which destroys bone homeostasis and causes bone density reduction and bone tissue microstructure destruction [8, 9]. Loss of bone mass will lead to osteoporosis, which may mean a decrease in bone strength. Therefore, steroid-associated ONFH and osteoporosis are closely related. Gangji et al. [10] measured the bone mineral density of the lumbar spine and femoral neck of non-traumatic ONFH patients and healthy control. They reported that ONFH is associated with low bone mineral density. Bone mineral density (BMD) is the most extensively used phenotype to evaluate bone strength [11]. However, Siris ES et al. [12] consider that only 50–70% of total bone strength can result from BMD, which means that bone density is not solely used to judge bone strength. Since compressive strength index (CSI) measures the capacity to withstand compressive forces proportional to bodyweight along the main femoral neck axis, it is considered to potentially improve the performance of the evaluation of hip bone strength [13]. Furthermore, the bending strength index (BSI) and the impact strength index (ISI) were calculated according to the average femoral neck width (FNW) and hip axis length (HAL), height, weight and femoral neck DXA-BMD [14]. Meanwhile, existing studies found that patients with the femoral head necrosis have active bone formation and bone resorption, and have obvious abnormal lipid metabolism [15]. Therefore, bone turnover markers can reflect the development of non-traumatic ONFH to a certain extent. This study hypothesized CSI, BSI, ISI and bone turnover markers as good indicators to evaluate the strength of the femoral head and predict the collapse of the femoral head necrosis. Therefore, this study aimed to explore the predictive effect of CSI, BSI, ISI and bone turnover markers on the collapse of the femoral head in steroid-associated ONFH patients.

## Materials and methods

### Patients

The nonoperative steroid-associated ONFH patients admitted to the First Affiliated Hospital of Guangzhou University of Chinese Medicine from January 2017 to June 2019 were retrospectively analyzed. The patient's ONFH was diagnosed by MRI scan.The inclusion criteria were defined as: (i) Association Research Circulation Osseous (ARCO) stage II steroid-associated ONFH patients, (ii) patients aged from 19 to 60 years, (iii) The patient has used corticosteroids for more than half a year and has now been stopped,(iv)patients receiving non-surgical treatment. Exclusion criteria were patients with continuous use of steroids, with a history of alcoholism, a history of a hip injury. Furthermore, patients with congenital hip diseases, tumors, and diseases that affect bone metabolism were excluded. All patients were and were divided into the collapsed group and the non-collapsed group according to whether the femoral head collapsed(femoral head collapse > 2 mm) [16]. This study was approved by the ethical review board of The First Affiliated Hospital of Guangzhou University of Chinese Medicine (No: Y[2019]118).This study obtained informed consent from all participants.

### Conservative treatment

All patients accepted the oral administrations with TCM *Yuanshi Shengmai Chenggu* tablet (6 tablets each time, 3 times per day, institutional approval number: Z20070828) and *Fufang Shengmai Chenggu* capsule (4 capsules each time, 3 times per day, and institutional approval number: Z20071224). The two drugs were prepared by the First Affiliated Hospital of Guangzhou University of Traditional Chinese Medicine (Guangzhou, China). Following the oral administration, muscle group exercises were performed with an emphasis on anterior flexor muscles, abductor muscles and adductor muscles and protective weight-bearing exercises.This treatment regimen was approved by the First Affiliated Hospital of Guangzhou University of Traditional Chinese Medicine.

### Measurements

**Fig. 1:**
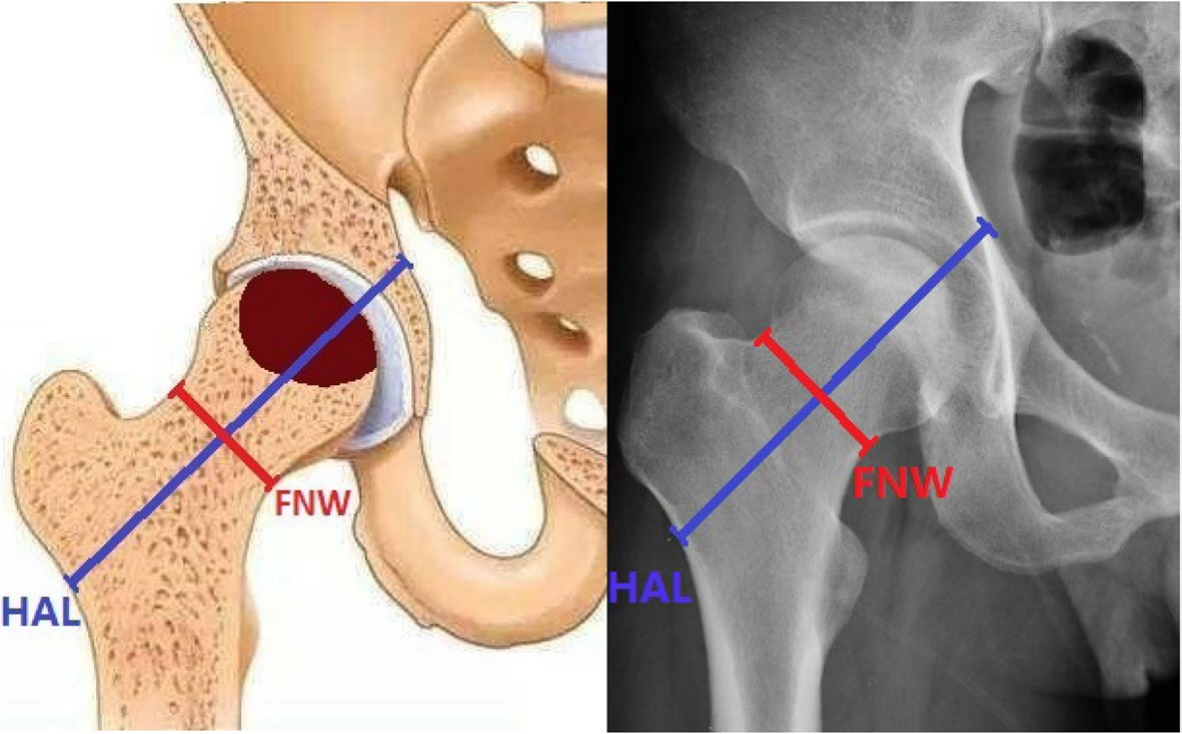
Geometry of the femoral neck: FNW: femoral neck width, HAL: hip axis length. The equations adopted included: CSI = (BMD*FNW) / weight; BSI = (BMD*FNW.^2^) / (HAL* weight); ISI = (BMD*FNW*HAL) / (height *weight)

All plain radiographs were taken by radiology technologists using standardized techniques. Standard anteroposterior (AP) views were obtained with the patients in the supine position and lower extremities internally rotated 15, centred on the line connecting the midpoint of the anterior superior iliac spine and the pubic symphysis. The film focus distance was 100 cm, and the X-ray beam was directed to the midpoint of the pubic symphysis.Hip x-rays magnification was 1.15. CSI, BSI and ISI at the site of femoral neck were calculated according to the mean FNW and HAL, together with height, weight and femoral neck DXA-BMD, by referencing the description of Karlamangla et al. [17]. The mean FNW was obtained from the 1.5 cm wide femoral neck region of interest (area (cm2)/1.5 (cm)). The HAL could indicate the distance along the femoral neck axis from the lateral margin at the base of the greater trochanter to the inner pelvic brim. The equations applied were: CSI = (BMD*FNW) / weight, BSI = (BMD*FNW2) / (HAL* weight), ISI = (BMD*FNW*HAL) / (height *weight) (Fig. 1).

### Biochemical markers of bone turnover test

At the time of first admition to the hospital,the patients serum was prepared after the centrifugation at 3,000 rpm for 15 min. Serum concentrations of P1NP, β-CTX, ALP, N-MID and 25(OH)D were measured with a Roche electrochemiluminescence system (Cobas E602; Roche Diagnostics, Basel, Switzerland).

### Statistical analysis

The statistical analysis was conducted with SPSS version 24.0 software (IBM Corp., Armonk, NY). All quantitative data were expressed as the mean ± standard deviation. The independent samples t-test was performed to compare the results achieved by two independent groups. The categorical data were analyzed by performing a chi-squared test. A receiver operating characteristic (ROC) curve was used to assess the cutoff point for CSI, BSI, ISI, t-P1NP and β-CTx. Computed as the sum of sensitivity and specificity minus 1, Youden’s index ranged from 0 to 1 (no diagnostic efficacy to perfect diagnostic efficacy). It was adopted to assess the performance of the mentioned diagnostic thresholds. Kaplan–Meier survival analysis was performed using the femoral head collapse as an endpoint.we use log-rank test to compare significance of two survival curves.A P value less than 0.05 was considered to show statistical significance.

## Results

### General information

A total of 62 patients with 86 hips were included here, 24 of whom had bilateral ONFH. These patients were taking corticosteroids due to diseases such as systemic lupus erythematosus, dermatomyositis, rheumatoid arthritis, and chronic lymphocytic leukemia.These patients took dexamethasone and prednisone for more than half a year, and the total dose was about 2500 mg-7300 mg.There was no statistically significant difference between the two groups in corticosteroids administration time and dose (*P* > 0.05).Their average age was 40.3 ± 9.82 years. The average follow-up period was 3.9 ± 0.5 years was achieved. 48 hips with ONFH with the femoral head collapse were recruited in the collapsed group. No statistical difference was identified in age, gender, height, weight, BMI, femoral neck BMD and JIC(Japanese Osteonecrosis Investigation Committee) classification between the two groups of patients (*P* > 0.05) (Table 1).

**Table 1:** Clinical characteristics of participants

Parameters	Collapse group (*n* = 48 hips)	Non-collapse group (*n* = 38 hips)	t /χ^2^	*P*-value
Age (years)	40.6 ± 9.14	39.9 ± 10.8	0.302	0.763
Gender, n (%)				
Male	12(35.3%)	11(39.3%)	0.105	0.746
Female	22(64.7%)	17(60.7%)		
Height(cm)	166.7 ± 7.43	165.3 ± 8.20	0.893	0.375
Weight(kg)	66.8 ± 13.7	65.1 ± 13.0	0.563	0.575
BMI(kg/m^2^)	23.9 ± 4.04	23.7 ± 3.86	0.187	0.852
Femoral neck BMD(g/cm^2^)	1.10 ± 0.13	1.11 ± 0.15	-0.354	0.724
JIC type,n(%)				
Type-A	0(0.0%)	0(0.0%)	0.204	0.903
Type-B	15(31.2%)	12(31.6%)		
Type-C1	21(43.8%)	18(47.4%)		
Type-C2	12(25.0%)	8(21.1%)		

### CSI, BSI, ISI and bone turnover markers in the non-collapse *v.s.* collapsed groups

The mean CSI in the collapsed group was significantly lower than that in the non-collapsed group (*P* = 0.011). The non-collapsed group achieved a significantly higher mean BSI than the collapsed group (*P* = 0.042). Moreover, the mean ISI of non-collapsed group was 0.49 ± 0.05, significantly higher than that in the collapsed group (*P* = 0.000). Specific to bone turnover markers, t-P1NP levels in the non-collapsed group were significantly higher than those in the collapsed group (*P* < 0.05). Furthermore, the non-collapsed group achieved significantly higher β-CTx levels than the collapsed group (*P* < 0.05). No statistical difference was identified in N-MID, 25(OH)D and ALP between the two groups (*P* > 0.05) (Table 2).

**Table 2:** Comparison of CSI, BSI, ISI and bone turnover markers between the non-collapse and collapse groups

Parameters	Collapse group (*n* = 48 hips)	Non-collapse group (*n* = 38 hips)	t	*P*-value
CSI (g/kg.m)	5.19 ± 0.95	5.93 ± 0.76	-2.686	0.011
BSI (g/kg.m)	2.09 ± 0.56	2.44 ± 0.493	-2.104	0.042
ISI (g/kg.m)	0.43 ± 0.04	0.49 ± 0.05	-4.130	0.000
t-P1NP (ng/mL)	57.2 ± 20.2	72.1 ± 20.1	-2.300	0.027
β-CTx (ng/mL)	0.51 ± 0.18	0.66 ± 0.22	-2.259	0.030
N-MID (ng/mL)	17.9 ± 6.27	17.8 ± 6.46	0.042	0.967
25(OH)D (ng/mL)	27.5 ± 9.22	27.1 ± 8.20	0.128	0.899
ALP(U/L)	68.3 ± 19.4	67.5 ± 18.5	0.625	0.517

### Multivariate logistic regression analysis for risk factors of the femoral head collapsed in ONFH patients

By employing significant factors obtained from the mentioned comparison, CSI, BSI, ISI, t-P1NP and β-CTx as independent variables, and the development of the femoral head collapsed as the dependent variable. In addition, an unconditional logistic regression analysis was conducted. As indicated by multivariate logistic regression analysis, CSI(*P* = 0.044), ISI (*P* = 0.001),t-P1NP(*P* = 0.048) and β-CTx (*P* = 0.003) were protective risk factors for the collapse of the femoral head in ONFH patients (Table 3).

**Table 3:** Logistic regression analysis odds ratio of CSI, BSI, ISI, t-P1NP and β-CTx

Parameters	B	S.E	Wals	*P* value	OR	95% CI
CSI	-1.261	0.627	4.047	0.044	0.283	0.083–0.968
BSI	0.430	1.015	0.180	0.671	1.538	0.210–11.236
ISI	-26.080	7.654	11.610	0.001	0.000	0.00–0.0.00
t-P1NP	-0.035	0.018	3.901	0.048	0.966	0.933–1.000
β-CTx	-5.356	1.822	8.638	0.003	0.005	0.000–0.168

### ROC analysis of CSI, BSI,ISI,t-P1NP and β-CTx

**Fig. 2:**
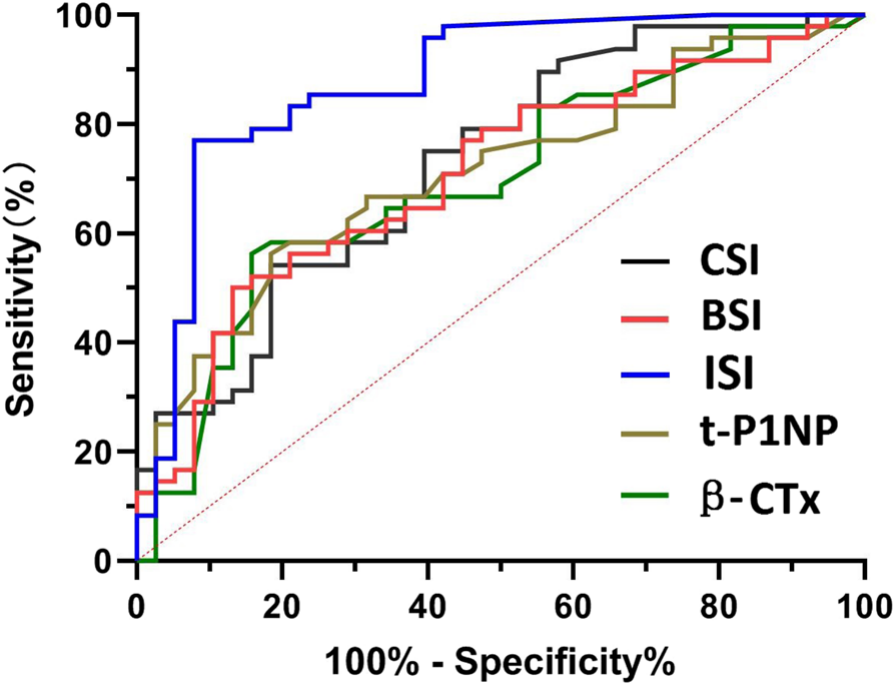
ROC curve analysis of CSI,BSI,ISI,t-P1NP and β-CTx model for prediction of steroid-associated ONFH collapse compared with non-collapse steroid-associated ONFH

As revealed by the results of ROC analysis, the cutoff point of CSI was 5.373 (sensitivity = 54.2%, specificity = 81.6%, *P* < 0.05). The cutoff point of BSI reached 2.007(sensitivity = 50.0%, specificity = 84.2%, *P* < 0.05). Moreover, the cutoff point of ISI was 0.435(sensitivity = 77.1%, specificity = 92.1%, *P* < 0.05). The cutoff point of t-P1NP reached 56.04(sensitivity = 56.3%, specificity = 81.6%, *P* < 0.05). Furthermore, the cutoff point of β-CTx was 0.471(sensitivity = 56.3%, specificity = 84.2%, *P* < 0.05) (Table 4). The area under the curve (AUC) values included CSI:0.729, BSI:0.707, ISI:0.878, t-P1NP:0.709 and β-CTx:0.696 (Table 4 Fig. 2).

**Table 4:** Area under the ROC curve

Parameters	Area	Standard error	*P* value	Progressive 95% CI		Cutoff value	Youden’s index
				Lower limit	Upper limit		
CSI	0.729	0.054	0.000	0.622	0.836	5.373	0.358
BSI	0.707	0.056	0.003	0.597	0.817	2.007	0.368
ISI	0.878	0.040	0.000	0.801	0.956	0.435	0.771
t-P1NP	0.709	0.055	0.005	0.601	0.818	56.04	0.379
β-CTx	0.696	0.057	0.008	0.583	0.808	0.471	0.405

### Survivorship analysis of CSI, BSI,ISI,t-P1NP and β-CTx

**Fig.3:**
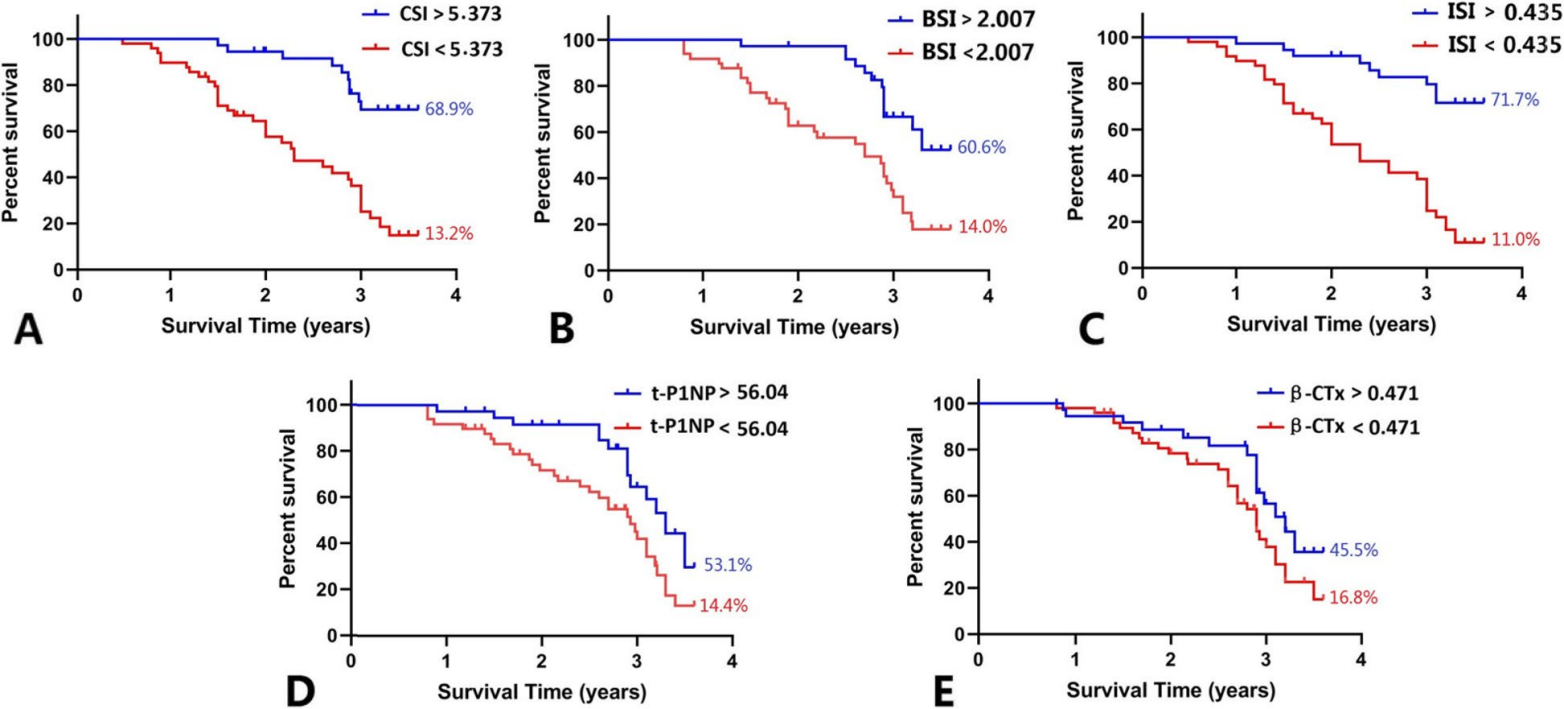
Survivorship analysis of CSI, BSI,ISI,t-P1NP and β-CTx

**Fig. 4:**
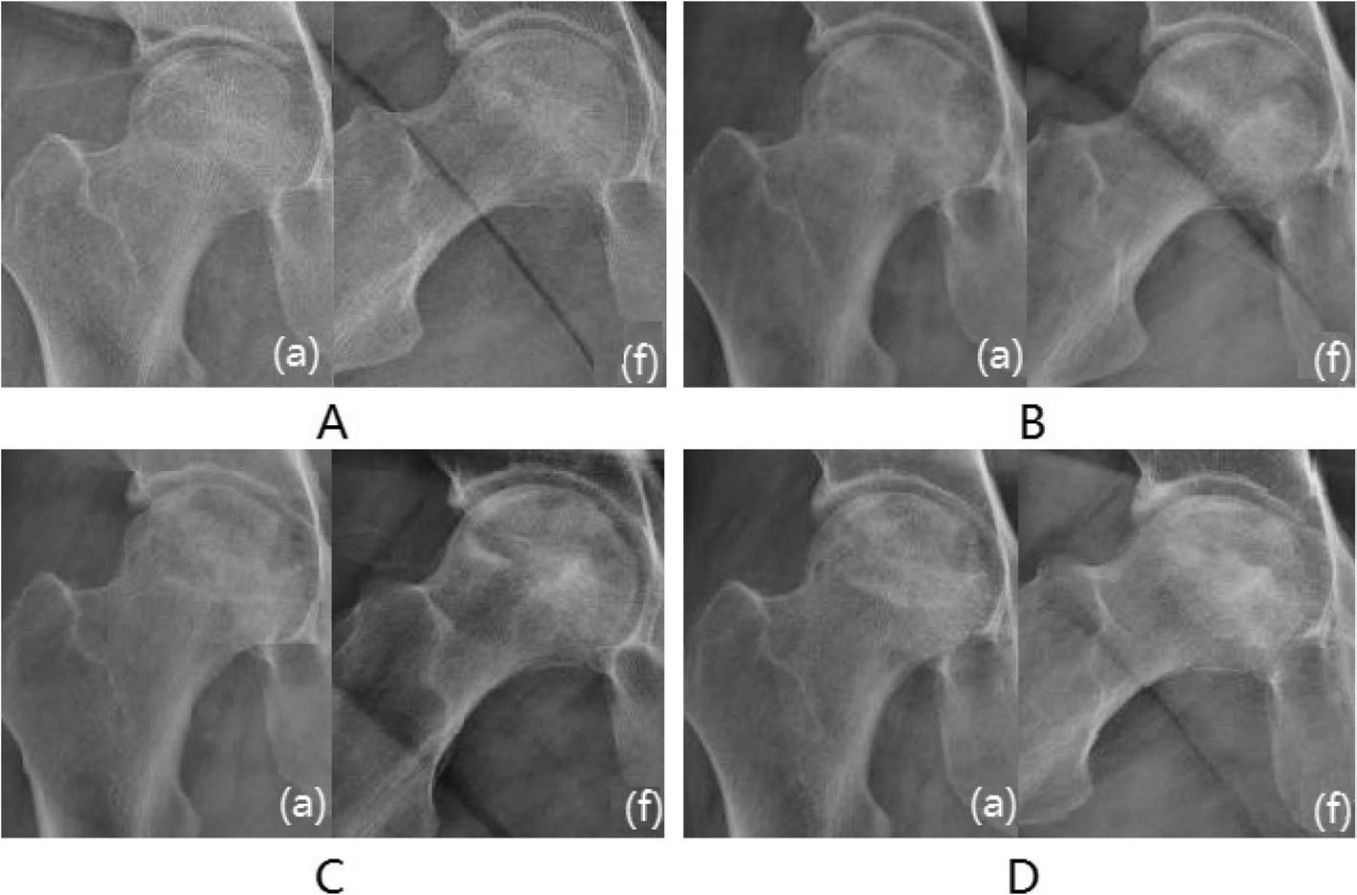
Typical case 1: Male, 45 years, with right hip pain for more than 8 months. **A **Radiograph imaging at an anteroposterior (a) and frog (f) lateral view. The patient's CSI, BSI and ISI were 6.36,2.36 and 0.53,respectively. **B **Six months. **C** 1 year. **D** After 2 years, the femoral head did not collapse significantly, and the density of the necrotic area increased

**Fig. 5:**
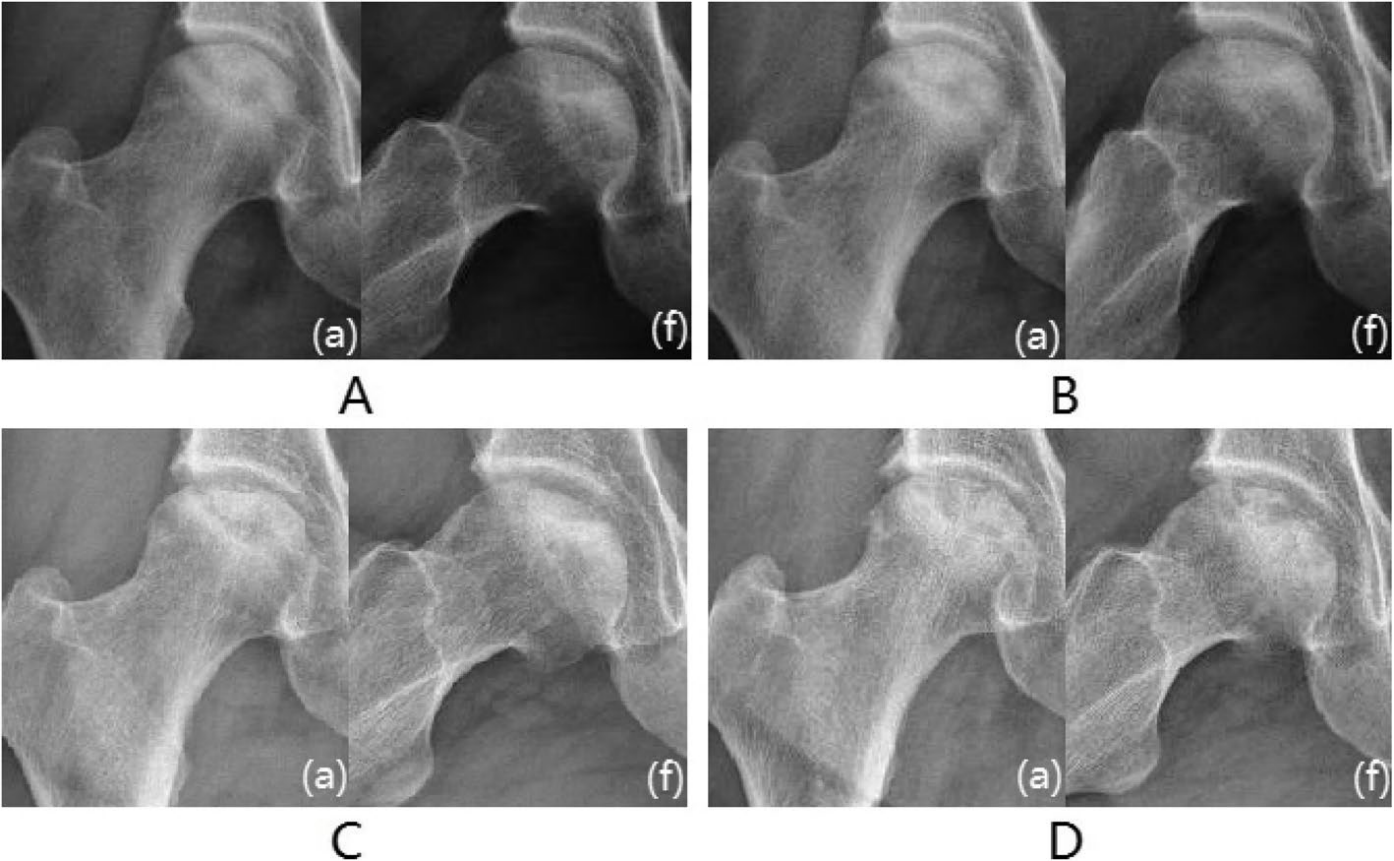
Typical case 2: Female, 46 years, with right hip pain for more than 11 months. **A **The patient's CSI, BSI and ISI were 4.84,1.63 and 0.41,respectively. **B **After half a year. **C **1.5 years. **D **After 2.5 years, the femoral head has become slightly flat and the joint space is acceptable

Survivorship analysis was conducted according to the cutoff value of the ROC curve. According to the survivorship analysis (with the femoral head collapse as the endpoint), the mean survival time of the hips of patients with CSI over 5.373 was greater (*P* < 0.05) than that of patients with CSI less than 5.373(Fig. 3A). According to the survival analysis, the probability of occurring the femoral head collapse within 3.5 years after conservative treatment was 39.4% for hips with BSI greater than 2.007(*P* < 0.05) (Fig. 3B).Moreover, the femoral head survival rate within 3.5 years after conservative treatment was 71.7% for hips with ISI greater than 0.435, 11.0% for hips with ISI less than 0.435(*P* < 0.05) (Fig. 3C). Furthermore, the femoral head survival rate within 3.5 years after conservative treatment was 53.1% for hips with t-P1NP greater than 56.04, 14.4% for hips with t-P1NP less than 56.04(*P* < 0.05) (Fig. 3D).However, no significant statistical difference was found in terms of the mean survival time between patients with β-CTx greater than 0.471 and patients with β-CTx less than 0.471(*P* > 0.05)(Fig. 3E). The clinical follow-up found that patients with the femoral head necrosis with large ISI and CSI had good conservative treatment effects and fewer femoral head collapses(Fig. 4).However, patients with the femoral head necrosis with less ISI and CSI were prone to the femoral head collapse(Fig. 5).

## Discussion

This study included 62 patients in total. The comparison was drawn on CSI, BSI, ISI and bone turnover markers. ROC curve was estimated to assess the sensitivity and specificity of CSI, BSI, ISI, t-P1NP and β-CTx in collapse prediction. As indicated by the mentioned results, ISI could effectively predict the femoral head collapse of steroid-associated ONFH patients.

Bone turnover markers are capable of reflecting the bone metabolism of the whole body and exhibit more sensitivity than bone density. β-CTx refers to a degradation product of collagen fibers in the extracellular matrix, which can indicate the degradation rate of the bone matrix and the activity of osteoclasts [18, 19]. t-P1NP is capable of indicating the synthesis rate of type I collagen, as well as the activity of osteoblasts [20]. ALP is largely synthesized by the liver and bone cells, and it can also reflect the activity of osteoblasts under the normal liver function [21]. 25(OH)D fundamentally helps maintain human skeleton [19]. As suggested by existing studies, the repair process of ONFH is similar to the mechanism of microfracture repair, which is a high rate of the bone turnover process [22]. As reported from this study, the non-collapsed group t-P1NP and β-CTx were significantly higher than the collapsed group. This result may be explained as the repair reaction occurs in the necrotic area after ONFH, and the early local bone metabolism is relatively active. With the disease progressing, the necrotic area will be absorbed and repaired, the local bone density will increase, and bone metabolism tends to be stabilized. The logistic regression analysis revealed that low t-P1NP and β-CTx were significantly associated with steroid-associated ONFH collapse. The AUC values of t-P1NP and β-CTx in this study were lower than ISI, suggesting that ISI is more effective in predicting steroid-associated femoral head collapse.

In normal scenarios, a dynamic balance exists between osteoblast-mediated bone formation and osteoclast-mediated bone resorption [23]. After the presence of steroid-associated ONFH, bone resorption and bone reconstruction will be triggered. New blood vessels and granulation tissue grow in the necrotic area, and the necrotic bone tissue tends to be absorbed and replaced by a new bone [24]. A study found that the bone mineral density of non-traumatic ONFH at ARCO stage I and II is lower than that at stage III and IV [10]. As reported from this study, no statistically significant difference was identified in bone mineral density between the collapsed group and the non-collapsed group, probably attributed to the selection of stage II steroid-associated ONFH patients in this study. As indicated by this result, early steroid-associated ONFH cannot predict the femoral head collapse through bone density. The different morphology of the proximal hip joint causes different mechanical conduction [25]. This study found that the non-collapsed group CSI, BSI and ISI were significantly larger than the collapsed group. CSI, constructed from structural engineering principles, measures the maximum compressing load that the femoral neck could withstand per unit thickness cross-sectional slice and the unit of normal compression load exerted on the femoral neck [22]. As indicated by the calculation formulas of CSI, BSI and ISI, when BMD, weight and height are identical, the larger the FNW, the larger CSI, BSI and ISI will be [23]. As suggested by the study, the narrower the femoral neck, the lower the bending resistance will be [26]. The wider the femoral neck and the larger the femoral head, make the more average the hip joint stress distribution will be, which is less prone to stress concentration on the femoral head surface [27]. Accordingly, the greater CSI, BSI and ISI, the femoral head in patients with ONFH will be less likely to collapse. As revealed by the ROC analysis results of this study, ISI is more effective than others in predicting steroid-associated ONFH collapse. ISI reflects the ability of the femoral neck to withstand axial compression and bending forces and absorb energy from impact [28]. Accordingly, ISI is capable of more comprehensively indicating the strength and mechanical conduction of the proximal femur.Early prediction of ONFH femoral head collapse has great clinical significance for treatment.At present, it is widely believed that hip-preserving surgery has a significant effect on patients in the early stage and before the femoral head collapses, but THA is often selected for cosmetic treatment of the collapsed femoral head [29].Non-avascularized bone grafting [30]and avascularized bone grafting [31] treatment of early (ARCO II) ONFH have better clinical effects.Therefore, for ONFH patients at high risk of collapse, we recommend early hip-preserving surgery.

The innovation of this study is that the composite indices of femoral neck strength are initially used to predict the steroid-associated ONFH femoral head collapse. However, this study also has several limitations. First, a clinical observational study was conducted here, and the conclusions should be further verified by biomechanics. In addition, this study was a single-center study with a limited number of cases. Whether the proposed method can achieve good consistency in other centers should be further explored.

In conclusion, the composite indices of femoral neck strength can predict the steroid-associated ONFH femoral head collapse more effectively than the bone turnover markers. ISI of 0.435 is a potential cut off. Based on the above data, future collapse of early steroid-associated ONFH can be predicted. 

## Data Availability

The datasets generated during and analyzed during the current study are not publicly available due to datasets involves patient privacy but are available from the corresponding author on reasonable request.
